# Long Biliopancreatic Limb Roux-En-Y Gastric Bypass Versus One-Anastomosis Gastric Bypass: a Randomized Controlled Study

**DOI:** 10.1007/s11695-023-06631-1

**Published:** 2023-05-13

**Authors:** Mohamed AbdAlla Salman, Ahmed Abelsalam, George Abdelfady Nashed, Mohamed Yacoub, Ahmed Abdalla

**Affiliations:** KasrAlainy School of Medicine, Cairo, Egypt

**Keywords:** Roux-en-Y gastric bypass (RYGB), One-anastomosis gastric bypass (OAGB), Biliopancreatic limb (BPL) length, Weight loss, Comorbidity remission

## Abstract

**Background:**

Roux-en-Y gastric bypass (RYGB) is the gold standard in bariatric surgery. The one-anastomosis gastric bypass (OAGB) procedure, first introduced by Dr. Rutledge, has demonstrated a 25% greater weight loss efficiency than the traditional Roux-en-Y gastric bypass (RYGB) procedure due to the substantially longer biliopancreatic limb (BPL).

**Aim of the study:**

The current work aimed to compare the outcomes of OAGB and long BPL RYGB regarding weight loss and comorbidity resolution.

**Patients and methods:**

This randomized controlled trial was done at our institution between September 2019 and January 2021. Patients who were candidates for bariatric surgery were randomly and equally allocated to two groups. Group A underwent OAGB, while group B underwent long BPL RYGB. Patients were followed up for 6 months postoperatively.

**Results:**

This study included 62 patients equally allocated to OAGB or long BPL RYGB, with no dropouts during follow-up. At 6 months, there was no statistically significant difference between the two groups regarding postoperative BMI (*P* = 0.313) and the EBWL (*P* = 0.238). There was comparable remission of diabetes mellitus (*P* = 0.708), hypertension (*P* = 0.999), OSA (*P* = 0.999), joint pain (*P* = 0.999), and low back pain (*P* = 0.999). Seven patients in the OAGB group experienced reflux symptoms (*P* = 0.011), which were managed by proton pump inhibitors.

**Conclusion:**

Extending the BPL in RYGB provides weight loss and comorbidity remission comparable to that of OAGB. Some OAGB-related reflux cases remain a concern. However, they were sufficiently controlled with PPIs. Due to OAGB superior technical simplicity, long BPL RYGB should be preserved for cases whom are more risky for bile reflux.

**Graphical Abstract:**

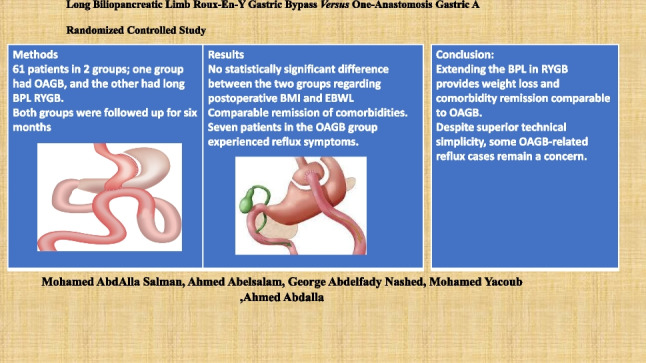

## Introduction

Obesity prevalence has been persistently increasing [1]. Bariatric surgery is the most effective long-term management strategy for morbid obesity [2]. Numerous techniques with various mechanisms of action have been documented throughout the development of bariatric surgery, historically classified as restrictive, malabsorptive, or a combination of both. This construct has no longer been considered. It is shown to be inaccurate with strong evidence that bariatric surgery entails neural and endocrine signaling pathways that affect the weight loss outcome [3].

For the past 20 years, Roux-en-Y gastric bypass (RYGB) has been the gold standard in bariatric surgery, and it is the second most frequently performed technique [4, 5]. This success has been attributed to its consistently achieving long-term weight loss, resolution of comorbidities, and acceptable mortality rates [6]. Typically, most individuals lose more than 70% of their excess weight within the first year following surgery [7].

Dr. Rutledge first introduced the one-anastomosis gastric bypass (OAGB) procedure in 199. The OAGB procedure features one anastomosis instead of two, resulting in technical simplicity, shorter healing times, and a lower incidence of early postoperative complications [8]. Additionally, the OAGB is simpler to modify or reverse [8–10].

Compared to the standard RYGB, one-anastomosis gastric bypass has shown about 25% greater weight loss efficiency, which is attributed to the malabsorptive impact of the substantially longer biliopancreatic limb (BPL) [11]. Additionally, longer BPL in RYGB has been linked to more considerable excess weight loss but similar postoperative morbidity [12].

The current work aimed to compare the outcomes of OAGB and long BPL RYGB regarding weight loss and comorbidity resolution.

## Patients and Methods

This randomized controlled study was conducted at our institution from September 2019 to January 2021. The study was conducted following the declaration of Helsinki and after approval of the institutional review board.

Adults with a BMI of ≥ 40 kg/m^2^ or ≥ 35 kg/m^2^ with comorbidities were suitable candidates for bariatric surgery, provided they tried nonsurgical treatment without success for at least 6 months. Patients who were candidates for RYGB or OAGB, fit for surgery, and accepted to participate were included.

All patients underwent the preoperative workup, including complete medical history, clinical evaluation, upper GIT endoscope, routine laboratory investigations, and abdominal sonogram. Patients with previous GIT surgery, congenital, inflammatory, or hemorrhagic GIT disease, hiatus hernia, advanced systemic disease, immunodeficiency, autoimmune connective tissue disorders, or significant mental or neurological conditions were excluded from the study.

Informed written consent was obtained from each patient before participation.

To assess obstructive sleep apnea (OSA), all patients were asked to complete the STOP-Bang questionnaire, which was developed for a concise OSA screening. It entails eight questions assessing snoring, tiredness, observed apnea, high blood pressure, BMI, age, neck circumference, and male gender. For each question, the patient scores 1 or 0 for “yes” or “no” answers, respectively. Therefore, the total score ranges from 0 to 8. The questionnaire has a high sensitivity using a cutoff score of ≥ 3 to diagnose OSA [13].

### Randomization

Patients were randomly allocated to two groups; group A underwent long BPL RYGB, and group B underwent OAGB. An independent hospital employer performed a straightforward randomization procedure using the closed envelope method.

### Operative Procedure

Preoperatively, the thromboembolic prophylaxis was ensured by subcutaneous low-molecular-weight heparin and compressive stocking. The surgical procedures were conducted as standardized under general anesthesia. In summary, pneumoperitoneum was induced, and then five trocars were inserted in the upper abdomen in a diamond-shaped pattern.

In the OAGB group, the gastric pouch was created. After identifying the Treitz ligament, a BPL length of 200 cm was created. A vertical or slightly oblique omega-loop, isoperistaltic, ante-colic, and side-to-side 30-mm gastrojejunostomy was performed.

In the RYGB group, an ante-colic ante-gastric RYGB procedure was performed laparoscopically on all patients. A 150-cm BPL was created and anastomosed side to side with the gastric pouch and pulled up in a connected ante-colic fashion. A 30-mm gastrojejunostomy was formed. Along the mesenteric border, the alimentary limb (AL), which was 75 cm long, was measured, and the entero-enterostomy was executed. Therefore, 225 cm of the small intestine was excluded.

A methylene blue test was used intraoperatively in both groups to check for any anastomotic leak. In some circumstances, a tube drain was retained.

### Postoperative Care and Follow-Up

Patients were encouraged to mobilize early following surgery, and the routine postoperative care was performed.

Patients were scheduled for follow-up appointments 1 week, 1 month, 3 months, and 6 months postoperatively, during which they underwent a complete clinical examination.

Excess body weight (EBW) and excess body weight loss (EBWL) were calculated as previously described [14].

Comorbidity resolution was evaluated according to the standardized outcome reporting of the American Society for Metabolic and Bariatric Surgery. Remission of type II DM, HTN, or OSA was considered if the patient discontinued all the medications with normal fasting glucose level (< 125 mg/dL), normal HbA1c (< 6.5%), and normal BP (< 120/80 mmHg). Improvement was defined as a reduction or cessation of the dosage of one or more medications but not necessarily all medications [15]. OSA remission was considered if the STOP-Bang score was ≤ 2 [13].

### Study Outcomes

The primary outcome was the difference between the studied groups in the EBWL. The secondary outcome was the rates of remission/resolution of comorbidities.

### Statistical Analysis

The analysis of patients’ data was performed using the SPSS statistical software (IBM Corp., Armonk, NY, USA), version 26. Numerical values were tested for normality, and a student *t* test was performed accordingly. Categorical values were presented as frequencies and percentages and compared using the Chi-square test and *Z* test for proportion as appropriate. A *P* value less than 0.05 was considered statistically significant.

## Results

**Fig. 1:**
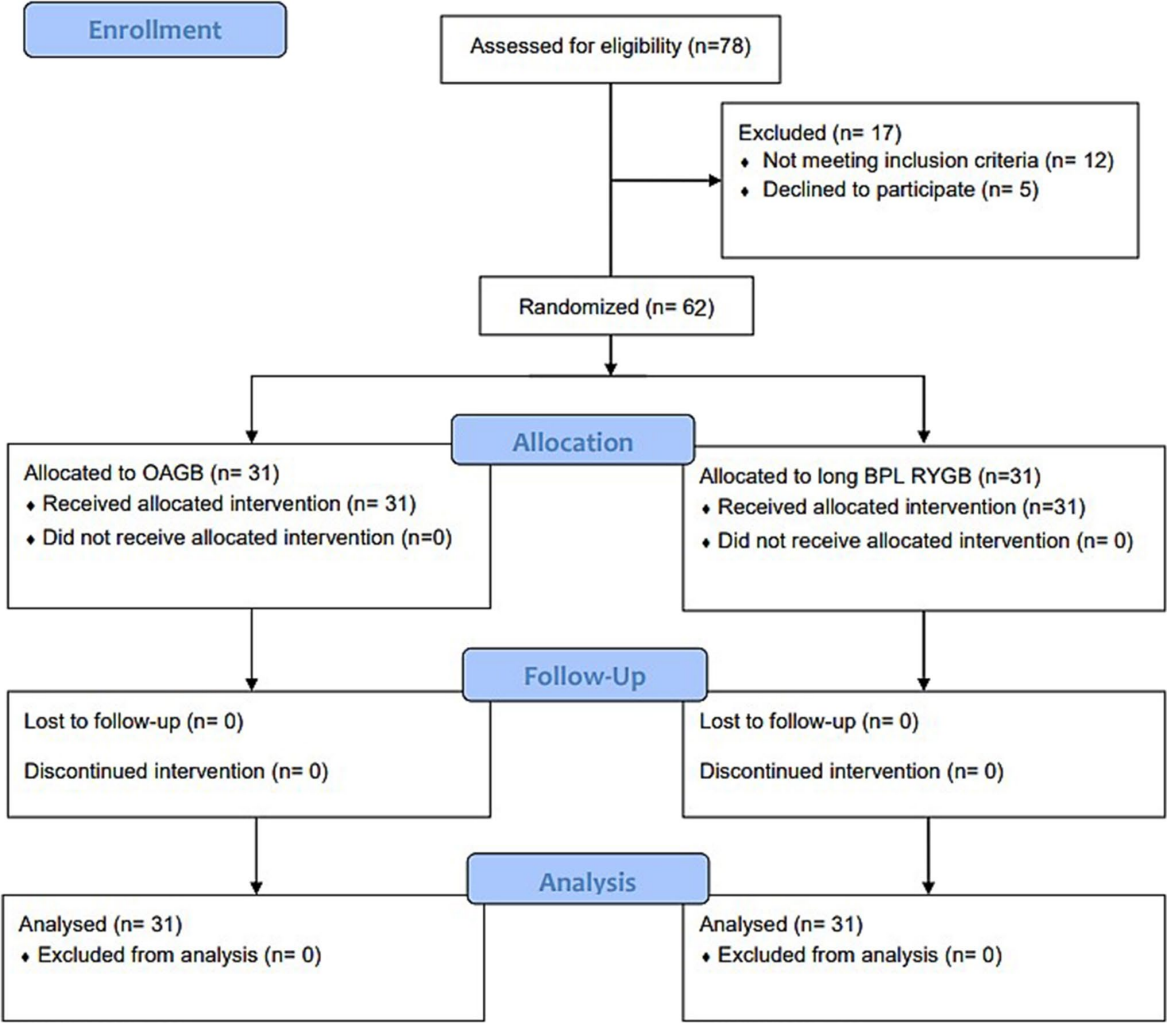
CONSORT flow diagram for the study patients

This randomized controlled study included 62 patients equally allocated to OAGB (*n*=31) or long BPL RYGB (*n*=31). All patients completed the study without dropout (Fig. 1). The age ranged from 19 to 59 years, with a mean of 36.45 ± 8.7 in the OAGB group and 36.68 ± 9.97 in the long BPL RYGB group. Females constituted about two-thirds (62.3%). Both groups were comparable regarding age (*P* = 0.746) and sex (*P* = 0.118) (Table 1).

**Table 1:** Baseline data of the study patients

	Type of surgical technique				
	SAGB		Long BPL RYGB		*P*
	Mean ± SD	Range	Mean ± SD	Range	
Age (year)	36.45 ± 8.70	21–58	35.68 ± 9.97	19–59	0.746^a^
Baseline weight (kg)	137.53 ± 23.28	100–185	137.77 ± 24.14	85–198	0.968^a^
Baseline height (cm)	167.97 ± 7.12	155–180	167.10 ± 8.53	151–184	0.664^a^
Baseline BMI (kg/m^2^)	48.80 ± 7.83	37–64.80	49.29 ± 6.82	36–61.8	0.795^a^
Ideal body weight (kg)	70.25 ± 6.4	56.6–81.00	69.84 ± 7.06	57–84.5	0.810^a^
EBW (kg)	66.99 ± 21.52	33–107	67.93 ± 20.8	27.24–118	0.861^a^
	Count	%	Count	%	
Sex					
Male	9	29.0%	15	48.4%	0.118^b^
Female	22	71.0%	16	51.6%	
Comorbidities					
Type II diabetes mellitus	13	41.9%	22	71.0%	0.021*^c^
Hypertension	11	35.5%	9	29.0%	0.587^c^
OSA: STOP-Bang score					
Low	19	61.3%	18	58.1%	0.574^b^
Intermediate	2	6.5%	5	16.1%	
High risk	10	32.3%	8	25.8%	
Joint pain	7	22.6%	11	35.5%	0.263^c^
Back pain	8	25.8%	6	19.4%	0.544^c^

a: *t* and *t* value of the Student’s *t* test; b: χ^2^, Chi-square test; c: *Z* and *Z* test for proportion; *: statistically significant

The baseline body mass index (BMI) of the OAGB group ranged from 37 to 64.8 kg/m^2^, with a mean of 48.8 ± 7.83 kg/m^2^, while in the long BPL RYGB group, it ranged from 36 to 61.8 kg/m^2^, with a mean of 49.26 ±6.82 kg/m^2^. The excess body weight (EBW) ranged from 33 to 107 kg, with a mean of 66.99 ± 21.52 in the OAGB group, while in the long BPL RYGB group, it ranged from 27.2 to 118 kg, with a mean of 67.93 ± 20.8. No significant differences were observed between the two groups regarding baseline BMI (*P* = 0.795) or EBW (*P* = 0.861) (Table 1).

At baseline, diabetes mellitus was significantly higher in the long BPL RYGB group than in the OAGB groups (71% vs. 41.9%, respectively, *P*= 0.021). No significant differences were reported regarding hypertension (*P* = 0.587), joint pain (*P* = 0.263), and low back pain (*P* = 0.544) (Table 1).

Regarding baseline STOP-Bang score, the OAGB group demonstrated patients with mild (61.3%), moderate (6.5%), and high OSA risk (32.3%). In the long BPL RYGB group, patients were at low risk (58.1%), moderate risk (16.1%), and high risk (25.8%) (Table 1).

### Six-Month Post-surgery Follow-Up

At 6 months, the BMI ranged from 26 to 51 kg/m^2^, with a mean of 36.39 ± 6.85 kg/m^2^ in the OAGB group, while in the long BPL RYGB group, it ranged from 26 to 48.7 kg/m^2^, with a mean of 38.04 ± 5.88 kg/m^2^. The excess body weight loss (EBWL) percentage in the long BPL RYGB group ranged from 23.3 to 94.6%, with a mean of 54.78 ± 18.14%, while in the OAGB group, it ranged from 26.1 to 90.6%, with a mean of 49.64 ± 15.31%. No significant difference was detected between the two groups regarding postoperative BMI (*P* = 0.313) or EBWL (*P* = 0.238) (Table 2).

**Table 2:** Six-month data of the study patients

	Type of surgical technique				
	SAGB		Long BPL RYGB		*P*
	Mean ± SD	Range	Mean ± SD	Range	
BMI (kg/m^2^)	36.39 ± 6.85	26–51.6	38.04 ± 5.88	26–48.7	0.313^a^
EBWL (kg)	35.47 ± 13.74	15–75	31.6 ± 8.02	18–63	0.180^a^
EBWL%	54.72 ± 18.14	23.3–94.60	49.64 ± 15.31	26.1–90.6	0.238^a^
	Count	%	Count	%	
Comorbidities remission					
Type II diabetes mellitus					
Significant improvement	5	38.5%	6	27.3%	0.708^b^
Complete resolution	8	61.5%	16	72.7%	
Hypertension					
Significant improvement	4	36.4%	4	44.4%	0.999^b^
Complete resolution	7	63.6%	5	55.6%	
OSA: STOP-Bang score					
Low	30	96.8%	31	100.0%	0.999^b^
Intermediate	1	3.2%	0	0.0%	
High risk	0	0.0%	0	0.0%	
Joint pain					
Significant improvement	7	100.0%	10	90.9%	0.999^b^
Complete resolution	0	0.0%	1	9.1%	
Back pain					
Significant improvement	6	75.0%	5	83.3%	0.999^b^
Complete resolution	2	25.0%	1	16.7%	

a: *t* and *t* value of the Student’s *t* test; b: χ^2^, Chi-square test

Concerning comorbidities, remission was evident in all diabetic patients in both groups. Significant improvement and complete resolution occurred in 38.5% and 61.5%, respectively, of the OAGB group compared to 27.3% and 72.7%, respectively, of the long LBP group, with no significant difference (*P* = 0.708). Similarly, all patients with hypertension showed postoperative remission. Considerable improvement and complete resolution occurred in 36.4% and 63.6%, respectively, of the OAGB group compared to 44.4% and 55.6%, respectively, of the long LBP group, with no significant difference (*P* = 0.999) (Table 2).

Regarding OSA risk, most patients (96.8%) in the OAGB group and all patients in the long BPL RYGB group (100%) turned out to be low risk (*P* = 0.999) (Table 2).

Joint pain remission was reported in all patients in both groups. Significant improvement occurred in all patients of the OAGB group and most patients (90.9%) of the long LBP group. Only one patient revealed complete resolution in the long LBP RYGB group, with no significant difference (*P* = 0.999). Additionally, all patients with low back pain demonstrated postoperative remission. Significant improvement and complete resolution occurred in 75% and 25%, respectively, of the OAGB group compared to 83.3% and 16.7%, respectively, of the long LBP group, with no significant difference (*P* = 0.999) (Table 2).

About one-quarter (22.6%) of the OAGB group experienced reflux symptoms, while no reflux symptoms were reported in the long LBP group (*P* = 0.011). These symptoms were controlled by proton pump inhibitors (PPIs).

No major complications or mortality were encountered during the follow-up.

## Discussion

Evidence of the BPL’s function in bariatric surgery is growing as the OAGB gains popularity. In bypass surgeries, such as OAGB and RYGB, the adjustment of limb length helps achieve the target BMI [16].

Many studies have been done to improve outcomes of bypass surgeries by investigating limb lengths.

In published studies of OAGB, there have been wide variations in BPL length. The BPL length when Rutledge originally described OAGB was 180 cm [8]. For their initial 200 patients, Carbajo et al. utilized a length of 200 cm. After that, the small bowel length was measured, and 250 to 350 cm length was used [10]. Several authors adjusted the limb length according to the patient’s BMI, often between 200 and 250 cm [17]. Piazza et al. utilized 240 cm for seven patients with a BMI of 59.4 kg/m^2^ and 180–200 cm as a standard range [18]. Mahawar [19] stated that nutritional issues could be entirely avoided by utilizing a typical limb length of 150 cm. In the current study, we attempted to ensure that the benefits of the OAGB procedure were not obtained at the expense of nutritional compromise by making the bypassed limb 200 cm in length. We closely followed up with the patients while monitoring their micronutrients, minerals, and vitamins.

The BPL and AL lengths in patients who underwent RYGB in this study were 150 cm and 75 cm, respectively. A recent meta-analysis showed that shorter ALs (40–100 cm) are as effective for weight loss as longer ones (130–150 cm). Therefore, we thought an AL length of 75 cm was reasonable. The impact of BPL elongation in RYGB has been previously studied. According to a study by Nergaard et al., the diverted-OAGB procedure, which utilizes a longer biliopancreatic limb (BPL) of 200 cm and a shorter alimentary limb (AL) of 60 cm, was found to be more effective in achieving weight loss compared to the traditional RYGB procedure, which uses a shorter BPL of 60 cm and a longer AL of 150 cm over a 7-year follow-up period.

Darabi et al. compared three groups: group 1 (BPL: 50 cm and AL: 150 cm), group 2 (BPL: 150 cm and AL: 50 cm), and group 3 (BPL: 100 cm and AL: 100 cm). After 1 year, no change was observed in the EBWL. However, after 3 years, the longer BPL group had a higher EBWL [12]. One RCT compared a BPL of 75 cm and an AL of 150 cm to a BPL of 150 cm and an AL of 75 cm. Four years following surgery, a significantly higher EBWL was observed in cases with extended BPL RYGB [20]. It has been demonstrated that an AL of 50 cm and a BPL of 200 cm effectively achieve significant weight loss and improvement in diabetes mellitus [16]. A longer BPL of 100–150 cm has a more substantial anti-diabetic impact than the typical length of 50–75 cm in diabetic individuals who underwent RYGB [21]. In a more recent RCT, a BPL length of 200 cm induced better loss of weight and HbA1C levels at 1 year than a length of 50 cm [22].

Numerous studies showed that OAGB outperforms RYGB. A recent meta-analysis of 11 trials demonstrated that OAGB has higher diabetes mellitus remission rates than RYGB and is linked to more significant weight loss at 5 years [23]. These findings could be attributed to the higher malabsorptive characteristics due to its longer BPL length [23].

In line, the elongation of the BPL during RYGP in the current study yielded comparable short-term weight loss and comorbidity remission in both techniques.

As far as we know, only three studies compared OAGB and long BPL RYGB. Two were retrospective [24, 25], and only one was an RCT [26] to determine if morbid obesity patients can benefit from lengthening the BPL in RYGB. In the RCT, the authors changed the ratio of AL to BPL so that the BPL was longer (150 cm) than the AL (60 cm). The impact of this modified RYGB was compared to that of OAGB. The findings showed that prolonged BPL in RYGB is as effective as OAGB in regulating comorbid diseases, such as diabetes mellitus, and losing excess weight. However, weight loss in the OAGB was significantly higher 1 year following surgery.

The second study reported no significant differences regarding postoperative BMI and percentage of total weight loss between OAGB and elongated BPL RYGB groups, which is consistent with our findings. However, the study revealed that the OAGB bypass group had significantly higher levels of parathormone and lower levels of hemoglobin, iron, calcium, and vitamin D. This could be due to using a BPL of 250 cm for OAGB, which increases the risk of a severe nutritional deficit.

Fouly et al. [25] stated that OAGB was superior to long BPL RYGB regarding BMI and excess weight loss at 3, 6, 12, and 24 months, with no differences regarding comorbidity resolution.

The mechanism that enhances weight reduction and diabetes mellitus remission in long BPL RYGB remains uncertain. Extended BPL in RYGB may alter bile acids and intestinal flora in addition to more accentuated stimulation of the distal bowel [16]. A longer BPL bypasses a larger portion of the jejunum, resulting in early nutritional malabsorption and considerable weight loss [27]. Due to various factors, including an insufficient mixing with digestive secretions, the bariatric procedure typically results in minor fat malabsorption [28, 29]. The direct routing of food to the ileum may impact food tolerance and, subsequently, eating habits. In addition, bypassing most of the foregut in patients with a lengthy BPL may change the hormonal and immunological profile. Recent investigations reported variations in the GI hormone profile [30]. Long BPL patients have a hormonal profile characterized by an increase in fasting and postprandial GLP-1 and a reduction in insulin [31]. According to a recent rodent study, longer BPL causes Roux limb hypertrophy, less glucose absorption but more utilization, and persistently elevated incretin levels. The anti-diabetic effect of BPL is mediated by these pathways [32].

It has been suggested that bariatric surgery-related hypertension remission is caused by reduced inflammatory reactions and improved insulin resistance that could lessen arterial stiffness and sodium reabsorption with subsequent normalization of blood pressure levels [33]. Additionally, an elevation of some gut hormones, such as GLP-1 and peptide YY, may cause a diuretic and natriuretic effect on the kidney, contributing to hypertension remission [34].

Although weight loss improves and, in some cases, resolves OSA [35], several metabolic pathways unrelated to weight or BMI have been implicated in OSA pathophysiology [36], including improved glycemic control and decreased systemic inflammation. A recent study reported fewer apneic–hypoxic episodes and significant neurohormonal alterations 3 weeks after metabolic surgery but without significant weight reduction [37].

The substantial improvements in joint and back pain reported in the current study could be explained by various factors, including increased physical activity, better subjective well-being and self-perception, and mechanical causes such as reduced muscle and joint load [38].

Bile reflux is the most crucial complication following surgery. It should be treated promptly to prevent further damage to the esophageal mucosa. Due to this complication, many surgeons avoid OAGB for bariatric surgery [39, 40]. However, in the current study, all patients with reflux were sufficiently controlled by PPIs.

The small sample size and short-term follow-up are possible limitations of this study. Nevertheless, our work provides new evidence regarding the comparability of OAGB and long BPL RYGB in weight loss and comorbidity remission. However, OAGB remains superior in terms of technical simplicity.

## Conclusion

Extending the BPL in RYGB provides weight loss and comorbidity remission comparable to that of OAGB. Some OAGB-related reflux cases remain a concern. However, they were sufficiently controlled with PPIs. Due to OAGB superior technical simplicity, long BPL RYGB should be preserved for cases whom are more risky for bile reflux. Further RCTs with larger samples and a longer follow-up are highly recommended.
